# Effects of predation and habitat structure on the abundance and population structure of the rock shrimp *Rhynchocinetes typus* (Caridea) on temperate rocky reefs

**DOI:** 10.1007/s00227-012-1994-6

**Published:** 2012-07-13

**Authors:** Nicolas C. Ory, D. Dudgeon, C. P. Dumont, L. Miranda, M. Thiel

**Affiliations:** 1 The Swire Institute of Marine Science, The University of Hong Kong, Pokfulam Rd, Hong Kong, People’s Republic of China; 2 School of Biological Sciences, The University of Hong Kong, Pokfulam Rd, Hong Kong, People’s Republic of China; 3 Facultad Ciencias del Mar, Universidad Católica del Norte, Larrondo 1281, Coquimbo, Chile; 4 Centro de Estudios Avanzados en Zonas Áridas (CEAZA), Coquimbo, Chile

## Abstract

Human disturbances, such as overfishing, may disrupt predator–prey interactions and modify food webs. Underwater surveys were carried out at six shallow-water reef barrens in temperate waters of northern-central Chile from October to December 2010 to describe the effects of predation, habitat complexity (low, medium and high) and refuge availability on the abundance and population structure of the rock shrimp *Rhynchocinetes typus* (Rhynchocinetidae), an important mesoconsumer on subtidal hard substrata. Three sites were within managed (restricted access) areas for fishermen, and three were unmanaged (open-access). Field observations and tethering experiments were conducted to examine the relationship between fish and shrimp abundances, and the relative predation rates on shrimps. Direct effects of predation on *R. typus* body-size distribution were examined from shrimps collected in the field and fish stomachs. The presence and the abundance of *R. typus* increased with habitat reef complexity and refuge availability. Shrimp abundance was negatively related to fish abundance in managed areas, but not in open-access areas, where shrimp densities were the highest. Also, predation rates and body-size distribution of shrimps were unrelated, although fish consumed more large shrimps than should be expected from their distribution in the field. *R. typus* occurred most often in shelters with wide openings, offering limited protection against predators, but providing potential aggregation sites for shrimps. Overall, direct effects of predation on shrimp densities and population structure were weak, but indirect effects on shrimp distribution within reefs appear to have been mediated through behavioural responses. Our study highlights the need to assess both numerical and behavioural responses of prey to determine the effects of predator loss on mesoconsumer populations.

**Electronic supplementary material:**

The online version of this article (doi:10.1007/s00227-012-1994-6) contains supplementary material, which is available to authorized users.

## Introduction

The loss of marine top predators due to overfishing may disturb entire food webs through trophic cascades (Pauly et al. 1998; Pace et al. 1999). However, the effects of such disturbances on intermediate consumers are hard to predict because species of a same trophic level can be affected differently by predators (McPeek 1998). An increasing literature has demonstrated the importance of top-down effects on predator–prey interactions, with decreasing stocks of large predators releasing prey from predation pressure (e.g. Myers and Worm 2003; Baum and Worm 2009; Eriksson et al. 2011).

Predation effects on prey may also be mediated by environmental factors, among the most important of which is habitat structure (e.g. Almany 2004; Grabowski 2004). Habitats of high structural complexity may permit prey to maintain higher abundance or species richness by reducing predation intensity (reviewed by Denno et al. 2005). Prey survival is enhanced in complex habitats because detection is more difficult and many refuges of different sizes are available (Eggleston et al. 1990; Canion and Heck 2009). The protective value of complex habitats, however, is also influenced by the identity and behaviour of prey and predators (Main 1987; Primavera 1997). An important task for ecologists is to estimate biotic (direct and indirect predation) and environmental (habitat structure) effects on predator–prey interactions (Denno et al. 2005) in order to better predict the consequences of predator loss on mesoconsumer prey.

Benthic prey without morphological defences against predators (e.g. juvenile crustaceans, many shrimp species) is usually associated to complex habitats with small refuges that are inaccessible to most large predators. Tethering experiments in the field have confirmed that the survival of such prey increases with refuge availability (Eggleston et al. 1990; Mintz et al. 1994). However, this dependence on protective habitats tends to be reduced as the prey organisms grow and develop morphological defences (Wahle and Steneck 1992). In habitats of low complexity, or where suitable refuges are limited, vulnerable prey may aggregate in large groups that reduce the risks posed by predators (O’Brien and Ritz 1988; Childress and Herrnkind 2001a) or environmental stress (Thiel 2011). For example, the rockpool prawn *Palaemon elegans* (Palaemonidae), which inhabits areas of hard substratum where burying is impossible, has a greater tendency to aggregate than the brown shrimp *Crangon crangon* (Crangonidae), which is associated with soft substratum and can hide by burying itself (Evans et al. 2007).

The largest individuals of smaller and vulnerable prey may be exposed to higher predation risk because (1) suitable refuges for their body size are limited, (2) they are more detectable (Greene 1986), or (3) they are preferentially selected by predators (e.g. Main 1985). Variations in predation risk in relation to body size and ontogeny have been intensively examined using lobsters as model animals (e.g. Herrnkind and Butler 1986; Wahle and Steneck 1992; Childress and Herrnkind 2001b), but have been less studied in shrimps. This is despite the fact that shrimps are the dominant prey of many predatory fishes in temperate coastal waters (e.g. Albers and Anderson 1985; Garrison and Link 2000) and an important link between primary producers and higher trophic levels (Edgar and Shaw 1995a).

The aim of this study was to examine predator–prey interactions, mediated by the habitat structure, between predatory fishes and their mesoconsumer prey. The model species was the rock shrimp *Rhynchocinetes typus* (Rhynchocinetidae), which is common at depths of 4–30 m on rocky reefs along the coast of Chile (Miranda and Kong 1970). *R. typus* plays an important ecological role as prey for many fishes (Caillaux and Stotz 2003; Medina et al. 2004), but also as a mesoconsumer that controls benthic assemblages by selective consumption of prey (Dumont et al. 2009, Dumont et al. 2011a, b). During ontogeny, *R. typus* males go through several developmental stages: typus (female-like), intermedius and robustus, the dominant terminal-moult stage (Correa et al. 2000). Robustus and intermedius individuals are usually larger than typus males and possess hypertrophied chelipeds and third maxillipeds, possibly making them more susceptible to fish predators.

More specifically, we investigated the relationship between *R. typus* and predatory fishes by testing the hypothesis that shrimp abundance increased with the complexity of the habitat, in particular the availability of small refuges. We also tested whether reduced predation pressure led to enhanced shrimp abundance by comparing sites where fishing activity was restricted with open-access unmanaged sites where, it was assumed, fish would be less numerous. These comparisons were supplemented by direct measurements of predation rates on shrimps tethered in the field. Finally, we used data from stomach content analyses of predatory fish to test for a relationship between preferred prey size and shrimp population structure, based upon the assumption that prey of the preferred body size would be relatively more depleted at sites with the highest predation rates and fish abundance.

## Materials and methods

### Study sites

**Fig. 1:**
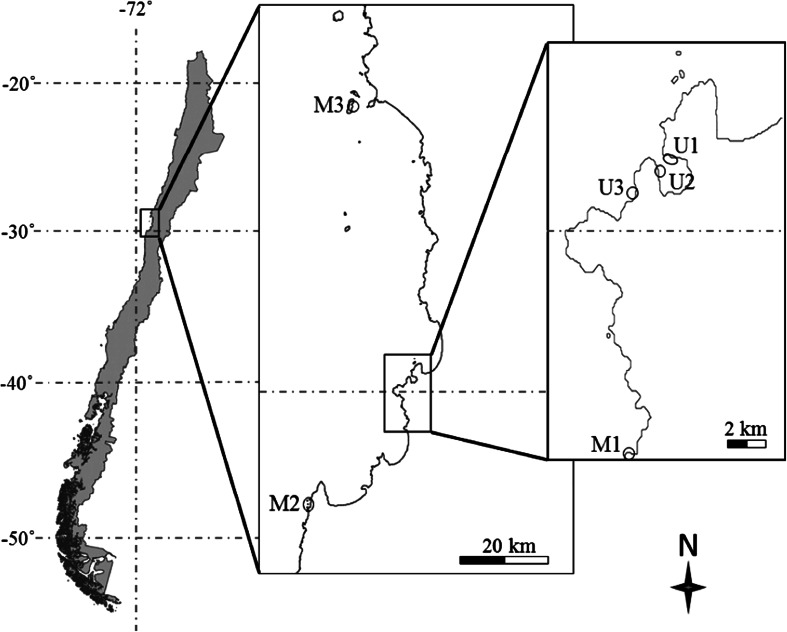
Map of Chile and insets of the geographical position of the six studied sites: OA1 (Herradura university: 9°58′S; 71°21′W), OA2 (Herradura Boca: 29°58′S, 71°22′W), OA3 (Guyacancito: 29°59′S, 71°22′W), MA1 (El Frances: 30°05′S, 71°22′W), MA2 (Caleta Totoral: 30°21′S, 71°40′W) and MA3 (Punta Choros: 29°15′S, 71°32′W)

This study was conducted in northern-central Chile, from October to December 2010 at 6 sites, separated by 1 to ~120 km (Fig. 1). Since we were interested in studying areas with differential predation pressure, we a priori selected sites that were assumed to have high abundances of fish predators and sites with suppressed numbers of predatory fishes. Three managed areas (MA1—MA3) were chosen because they are ‘management and exploitation areas’ (Castilla 1994), where access is limited to local fishermen only. Managed areas may have higher fish abundance compared to open-access sites, as shown for sites elsewhere along the Chilean coast (Gelcich et al. 2008) or in other parts of the world (McClanahan et al. 2006). MA1 is about 15 km away from the major city Coquimbo, while the two other sites were located close to small fishing villages (called ‘caletas’). Three open-access, unmanaged, sites (OA1—OA3) were located, respectively, inside, at the entrance and outside of La Herradura Bay, near Coquimbo.

All sites were located at a depth range of 4–7 m, on hard substrata, where the overgrowing community was dominated by encrusting corallines, and lacking foliose algae (Caillaux and Stotz 2003). The substratum was mostly bedrock, or compacted rocks covered by boulders and rocks of various sizes. The black sea urchin *Tetrapygus niger* (Arbaciidae) and *R. typus* dominate the guild of macroinvertebrate mesoconsumers (Dumont et al. 2011b). Shallow barrens of northern Chile were described to be of lower architectural complexity than deeper hard substrata that are usually dominated by kelp beds (*Lessonia trabeculata*) or suspension-feeders (e.g. the barnacle *Austromegabalanus psittacus*, the mytilid *Aulacomya ater*, and tunicates such as *Pyura chilensis*), which increase habitat complexity (Caillaux and Stotz 2003; Villegas et al. 2008).

### Habitat structure

The physical components of the reef structure were assessed after counting the shrimps from the same quadrat (see below). Measurements were taken directly in situ and from the analysis of photoquadrats (Table 1), taken with a digital camera Canon G11, using the freeware Image J^®^ (http://rsbweb.nih.gov/ij/).

**Table 1:** Explanatory variables used to identify the factors determining the presence and the abundance of *R. typus* within quadrats and fish abundance within transects on each of the six reefs

Explanatory variables	Method of measurement	Categories/values				
		1	2	3	4	5
Dominate substratum (% cover)	Photoquadrat	0–19	20–39	40–59	60–79	80–100
Rugosity of the reef surface (% irregular surface), adapted from Gratwicke and Speight (2005)	In situ and photoquadrat	<5	5–25	26–50	51–75	>75
Vertical height: distance from the lowest to the highest point of the reef (cm)	In situ and photoquadrat	0–9	10–19	20–39	40–75	>75
Number of refuge per quadrat	In situ	0	1	2	> 2	
Urchin presence	In situ	Absence, presence				
Site management		Open-access area (OA), managed area (MA)				
Fish abundance	In situ	Individuals 100 m^−2^				
Predation rates	In situ	Shrimp mortality site^−1^ (%)				

#### Reef composition

Four different types of substrata were recorded: bedrock or consolidated rocks, large boulders (average diameter >50 cm), medium boulders (20–50 cm) and small boulders (5–19 cm). Preliminary observations indicated that gravels and sand represented <5 % of the total substratum, and therefore, these were not considered for further analyses. The percentage cover of each substratum type in each quadrat was quantified from photoquadrats with the Coral Point Count with Excel extensions (CPCe) program using 100 random points (Kohler and Gill 2006). The substratum with the greatest percentage cover in each quadrat was used as an independent variable in the analysis of shrimp presence and abundance. Five quadrats in which two substrata had identical percentages were excluded, leaving a total of 327 replicate quadrats for the analysis.

#### Substratum architecture

Reef structural complexity is generally correlated with the rugosity of the reef (reviewed by Knudby et al. 2007), defined as ‘changes in the degree and direction of relief’ (Dunn and Halpin 2009), thereafter referred to as ‘irregularities’ of the reef surface. We visually assessed the rugosity in the field and from the photoquadrats using categories adapted from Gratwicke and Speight (2005) (Table 1). The score 1 of the rugosity index corresponds to a quadrat consisting of a flat substratum with <5 % irregularities of its surface. Quadrats with 5–25, 26–50 or 51–75 % of the substratum with irregularities received scores of 2, 3 and 4, respectively. The maximum rugosity score of 5 (Gratwicke and Speight 2005), which corresponds to a highly complex three-dimensional architecture, for example, assemblages of branching or digitate corals, was not observed at any of the sites included in this study. Reef height, defined as the distance from the bottom to the highest point of the reef within a quadrat (Wilson et al. 2007), was visually measured in situ, using the transect line (1-cm accuracy) as reference. However, because reef height was correlated with rugosity (Pearson correlation: *r* = 0.84, *P* < 0.01, *N* = 327), the analysis of the effects of substratum architecture on shrimp presence/abundance focused on rugosity only.

### Refuges

We followed the definition by Alexander et al. (2009), who defined a refuge as ‘(1) the meeting of 3 planes of the substratum with 1 of the planes forming an angle with the other planes of <90°, (2) the meeting of 2 planes of the substratum at an angle of 45° or less, and (3) the refuge must be deeper than the minimum dimension of its aperture’. A crevice differs from a hole in that it does not fully enclose the prey hiding inside and may offer less protection to the prey. We counted the total number of crevices and holes within a quadrat as 0, 1, 2, >2 refuges; quadrats with >2 refuges were pooled into a single category to ensure a frequency of >5 observations per category for each response variable (Zar 1999).

The aperture width of all refuges observed inside a quadrat was defined by the length of its smallest dimension using four categories (adapted from Alexander et al. 2009); refuges with an aperture of 1–5 cm fully enclose the prey and prevent the access of most adult predatory fishes in the study area; refuges with an aperture of 6–15 cm enclose the prey and may prevent access of many adult fishes but not of juvenile fishes (Ory personal observations). Refuges with an aperture of 16–30 cm are effective only against the largest fishes (e.g. the kyphosid *Graus nigra* >75 cm total length), but allow shrimps to aggregate. Holes or crevices with an aperture >30 cm do not protect shrimps from fishes, but may reduce their detectability if shrimps remain in the shadows; they also allow aggregations of large groups of shrimps. Categories of refuge aperture width were visually defined in situ using the graduated transect line as reference.

We visually counted the number of the sea urchin *T. niger* within each quadrat to examine whether they would influence the presence or abundance of *R. typus*. The smaller shrimps may gain protection from predators by taking refuge among the urchin, as reported for other shrimp species (e.g. Castro 1971; Criales 1984).

### Abundance of shrimps and fishes

To assess the abundance and diversity of predatory fishes, an underwater visual census (UVC) was conducted using six to eight 20-m random transects separated from each other by 10 m. All carnivorous fishes >5 cm total length (TL) observed within 2.5 m on each side of the transect line were counted and identified to the species level by the same SCUBA diver swimming at a constant speed of 5 m min^−1^, fast enough to reduce the risk of double-counting mobile fishes from one transect to another (Lincoln Smith 1988). Fish size was visually assessed using 5 size classes: 5–10, 11–20, 21–30, 31–40 and >40 cm TL. The UVC method tends to underestimate fish densities (Murphy and Jenkins 2010) and was used here as a basis to compare fish abundances between sites (McCormick and Choat 1987).

The abundance of shrimps was assessed along the same transect line by a second diver following the fish observer 5 min later, using a quadrat (50 × 50 cm) that was alternately placed at 2-m intervals on each side of the transect (i.e. 11 replicate quadrats per transect). Shrimps were counted up to 50 individuals; for higher densities, a conservative estimate of >50 individuals was used because of the risk of double counting the same individuals. The number of shrimps in the open (not associated with a refuge) was also counted.

### Intensity of predation on shrimps

The relative intensity of predation on shrimps was examined using tethering experiments in situ, along the same transect line, >1 h after fish and shrimp surveys. Individual shrimps were tethered with a transparent nylon monofilament tied around their body, in the gap between the cephalothorax and the first abdominal segment. The tethered shrimps were temporarily attached to individual rocks in aquaria with food ad libitum for a 24-h period prior to the experiment. All monofilaments remained successfully tied around the body of the shrimp, and after 24 h all tethered shrimps looked healthy and had similar behaviours to non-tethered shrimps.

The tethered shrimp were transported to each site in a cool-box (150 × 50 × 50 cm) filled with sea water. Just before diving, shrimps were transferred into individual plastic boxes (10 × 7 × 5 cm). Each shrimp was then attached with the monofilament tied to their body to the centre of a grey PVC plate (50 × 50 × 0.5 cm). Shrimps could not leave the plate and had nowhere to hide. Each plate was held in place with two 1-kg diving weights on open areas of the reef surface. Shrimps (18–31 individuals, depending on the site) were 10 m apart, and each individual was tethered only once. One of the tethered shrimp at each site was randomly chosen for video-recording using a Sony HDR-CX560V Camcorder in an underwater housing. All trials at one site were run the same day between 0900 and 1300 h. The percentage of predation on tethered shrimps was determined after 30 min; preliminary experiments showed that after 30 min >50 % of the tethered shrimp may be eaten. A missing shrimp was considered as a predation event if the monofilament had been cut or if shrimp remain were present on the intact loop (see also Herrnkind and Butler 1986).

### Body-size distribution of shrimps

#### Natural population

To compare the size distribution of *R. typus* between sites, shrimps were captured using an airlift sampler (for detailed description see Correa and Thiel 2003), with collection bags made of 4-mm nylon mesh. At each site, three to five random samples were taken on the same day. After collection, shrimps were kept in a large tank with running seawater and food ad libitum. The following day, pictures of all shrimps were taken using a 20× zoom USB microscope (Veho VMS-001). The carapace length (referred to as ‘size’ hereafter) of the shrimps was then measured using the freeware Image J^®^. We only measured sexually mature shrimps with a size >8 mm as smaller individuals could not always be sexed and due to the mesh size of the collection bags would have been under-represented in the samples.

#### Shrimps in fish stomachs

Predatory fishes were collected at three different sites (OA3, MA1 and MA2) by skin-diving fishermen using spear-guns. The aim was not to describe the natural diet of the fish, but to describe the relative predation rates on *R. typus* and to compare the sizes of shrimps consumed by some of the most common predators in the area.

Fish stomach contents were examined to assess the frequency of occurrence and the size of *R. typus* found inside. Fishes were identified to the species level, the total length (TL) measured to the nearest 0.5 cm, and the stomach removed and placed in a plastic container in a 10 % formalin solution. The number of *R. typus* present in each stomach was quantified from whole individuals or identified body parts (De Melo 2007). In the case of identical paired-appendages found apart from the shrimp body, we assumed the presence of two shrimps when we found more than two identical 3rd maxillipeds or pereopods. Morphometric measurements were taken from images using Image J^®^.

### Statistical analyses

#### Factors influencing shrimp and fish abundances

Generalized estimated equations models (GEE) were developed using the software SPSS^®^ v18 to examine the effect of the different components of the reef structure, the protected status of the sites (managed and open-access areas), predatory fish abundance and the relative predation rates on shrimp (i.e. shrimp mortality) on the presence or abundance of *R. typus* (Table 1). GEEs were chosen over generalized linear models because they account for autocorrelation that may occur between adjacent quadrats within a transect (Ballinger 2004). We used a working correlation matrix with an AR-1 structure (i.e. first-order autoregressive relationship) since the correlation between quadrats should decrease with the distance (Liang and Zeger 1986). Transects and quadrats were entered as dependent within-subject factors in the GEE model. The six study sites were separated by several kilometres, and spatial autocorrelation was improbable; they were thus entered as independent between-subject factors. The effect of each response variable on the regression model was tested with a Wald chi-square test. To avoid multicollinearity between explanatory variables, one of each pair of variables with correlations of *r* > 0.8 (Farrar and Glauber 1967) was excluded from the initial model.

The GEE model was fitted with a binomial distribution linked to a logit function to test the effects of the explanatory variables on the binary dependent variable ‘presence’ (i.e. 1) and ‘absence’ (i.e. 0) of *R. typus* within a quadrat. The null hypothesis that shrimp presence was explained by chance (50 % chances being present or absent; *P* = 0.5) was further tested using a chi-square goodness of fit for each category of the significant explanatory variables of the regression model (Zar 1999). Fish abundances and shrimp mortality were pooled in 3 categories each (respectively, <1, 1 and 2 individuals site^−1^ and <10, 11–15 and >15 % mortality site^−1^) to ensure >5 observations per category (Zar 1999).

The GEE model was also fitted with a negative binomial distribution linked with a log function to test the effects of the predictor variables on the abundance of *R. typus* within quadrats that had at least one shrimp. The negative binomial function best responded to the overdispersed distribution of the data (Gardner et al. 1995). A Dunn’s post hoc test was used to test the null hypothesis of similar shrimp densities among the categories of each significant explanatory variable yielded by the regression model.

GEE models were developed following a forward stepwise regression procedure with all explanatory not intercorrelated variables (Quinn 2002). The explanatory variables with α-levels greater than 0.05 were removed from the model. Interactions between the significant explanatory variables were then added to the model. Predictor variables are usually strongly correlated with their interaction terms and were centred by subtracting their mean for each observation (Quinn 2002). The final model chosen was the one with the lowest Quasi-Likelihood under the Independence model Criterion value, QIC (Pan 2001), equivalent to the Akaike’s Information Criterion (AIC) for repeated measures. We also assessed the validity of the model by applying a Wald-Wolfowitz test on the residuals to verify their randomness (Chang 2000).

A chi-square goodness of fit was used to test the null hypothesis that shrimp presence did not differ between small refuges (≤15 cm) and large (>15 cm) refuges. The spatial distribution of *R. typus* was tested with a Morisita’s index of aggregation, *I*_*d*_, which is based on counts of the total number of shrimps per quadrat. An *I*_*d*_ > 1, assessed with a chi-square test, indicated an aggregated dispersion (Brower et al. 1990).

In contrast to shrimp abundance, the abundance of large mobile predator fishes was assessed at the scale of the transect rather than at the quadrat scale. Accordingly, we built a GEE model, in which ‘transects’ were entered as dependent within-subject variable and ‘sites’ as independent between-subject variable, to test the effects of habitat structure and management status on fish abundance. The GEE model was fitted with a negative binomial distribution linked with a log function. The correlation matrix was AR-1. At the scale of the transect, the mean values of rugosity, number of refuges and dominant substratum indexes were strongly correlated. Hence, the structure of the habitat as described by rugosity only was incorporated in the GEE model.

#### Intersite differences between fish and shrimp abundances and predation rates

The abundances of shrimps and fishes, and fish sizes were compared among sites using Kruskal–Wallis tests since data were not normally distributed. We likewise tested whether shrimp abundance differed between managed areas and open-access areas. Dunn’s post hoc multiple comparisons test was also undertaken when appropriate, using Statistica^®^ v7.

We used a chi-square goodness of fit to test the null hypothesis of no difference in the proportions of shrimps present at the end of the tethering experiment (i.e. mortality). If the null hypothesis was rejected, post hoc comparisons were run to test for differences in observed proportions of shrimp presence between each pair of sites (Zar 1999). Spearman correlation was used to test whether mortality was related to fish abundance and reef structure (rugosity and number of refuges).

#### Shrimp sizes in the habitat versus in fish stomachs

Differences in *R. typus* median sizes among all sites were tested using Kruskal–Wallis tests. When appropriate, Dunn’s post hoc multiple comparisons tests were used to compare shrimp median sizes between sites.

Measurements of *R. typus* captured in the field indicated that shrimp size was strongly correlated with the length of the rostrum (RL), the length of the telson (TeL), the length of the propodus of the first pereopod (PL) and the last article of the third maxilliped of females and typus males (ML). Ratios between shrimp sizes and RL, TeL and PL were then calculated and used to assess the size of the shrimps found in fish stomachs when the carapace was missing or too degraded to be measured, but other body parts were found (Table S1).

At MA1, only five fishes could be captured. The replication was thus too low to analyse the size distribution of *R. typus* consumed by fishes at this site. These data were, however, pooled with the other two sites (OA3 and MA2) for the evaluation of the overall frequency of occurrence of *R. typus* in fish stomachs.

The size of the shrimps found in fish stomachs was pooled into four size categories (8.0–11.9, 12.0–15.9, 16.0–20.0 and >20 mm CL), each of which had a minimum of five individuals per category (Zar 1999). We used a chi-square goodness of fit to test the null hypothesis that the size distribution of *R. typus* consumed by fishes did not differ from the size distribution of the shrimps observed in the field. When the null hypothesis was rejected, tests of pairwise differences between observed and expected frequencies for each size category were undertaken. We also examined the relationship between fish size (TL) and the number and average size of the shrimps found in each fish stomach using a Spearman correlation.

## Results

### Influence of habitat structure on shrimp presence, abundance and spatial dispersion

**Fig. 2:**
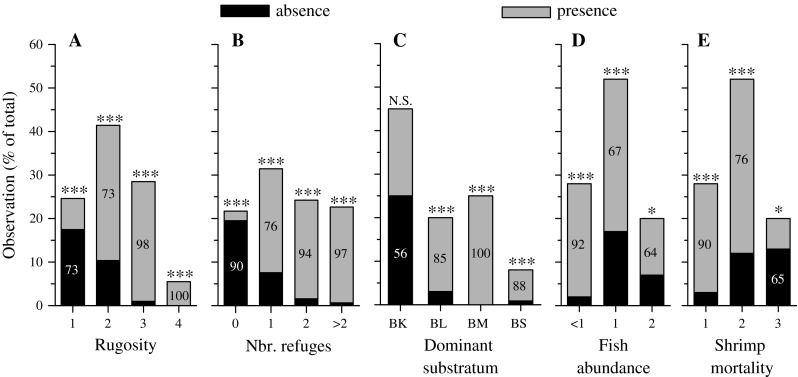
Frequency of the presence or absence of *R. typus* (% of the total observations; *N* = 327) in function of **a** the rugosity of the reef surface, **b** the number of refuges, **c** the dominant substratum type per quadrat (BK: bedrock; BL, BM and BS: respectively large, medium and small boulders), **d** fish abundance and **e** shrimp mortality (i.e. predation rates). *One and three asterisks* indicate that the frequencies of shrimp presence and absence differ (*P* < 0.05 and *P* < 0.001, respectively); *NS* indicates non-significance

The best model (fitted by the binomial logit GEE) indicated that the presence of *R. typus* within the quadrats was related to fish abundance and shrimp mortality (see below), rugosity, number of refuges and type of dominant substratum (Table 2A). Urchin presence did not influence that of shrimps, which were never observed beneath the spines of *T. niger*. Shrimps were generally absent in quadrats with the lowest rugosity and more often present in quadrats associated with a high rugosity (categories 2 and 3, Fig. 2a; Table S2). Similarly, they were mostly absent from quadrats without refuges, but present in quadrats with one or >1 refuges (Fig. 2b; Table S2). As a result, the presence of shrimps was positively influenced by large, medium and small boulders (Fig. 2c; Table S2). Shrimps were more often recorded in quadrats at sites where fish abundance and predation rates were low, but the proportion of empty quadrats increased when these two factors were higher (Fig. 2d, e; Table S2). At the site with the highest predation rate (MA1), shrimps were more often absent than present in quadrats.

**Table 2:** Maximum likelihood of the final GEE models testing the effects of the predictor variables on (A) the presence of *R. typus* within all the quadrats (goodness of fit of the final best-fitting model: QIC = 166.10, *N* = 292), (B) the abundance of *R. typus* within quadrats with >1 shrimp (QIC = 83.34, *N* = 205) and (C) fish abundance within transects (QIC = 31.34, *N* = 30), among all sites

Parameters	Wald chi-square	*df*	*P*
(A) *R. typus* presence (1), absence (0; reference) quadrat^−1^			
Dominant substratum	149.19	3	<0.001
Rugosity	7.70	3	0.021
Number of refuges	33.37	3	<0.001
Fish abundance	6.31	1	0.012
Predation rates	32.72	1	<0.001
Urchin presence^a^	1.42	1	0.234
(B) *R. typus* abundance (number of shrimp quadrat^−1^)			
Dominant substratum	20.774	3	<0.001
Number of refuges	13.139	3	0.004
Rugosity	8.215	3	0.016
Fish abundance × management status	4.77	1	0.030
Predation rates^a^	0.33	1	0.582
Fish abundance^a^	3.06	1	0.084
Fish abundance × rugosity^a^	1.91	3	0.591
Urchin presence^a^	0.05	1	0.824
(C) Fish abundance (number of fishes transect^−1^)			
Management status	0.07	1	0.798
Rugosity	4.87	3	0.087
Management status × rugosity	0.23	1	0.63

^a^Not included in the final model. Values displayed from initial models

**Fig. 3:**
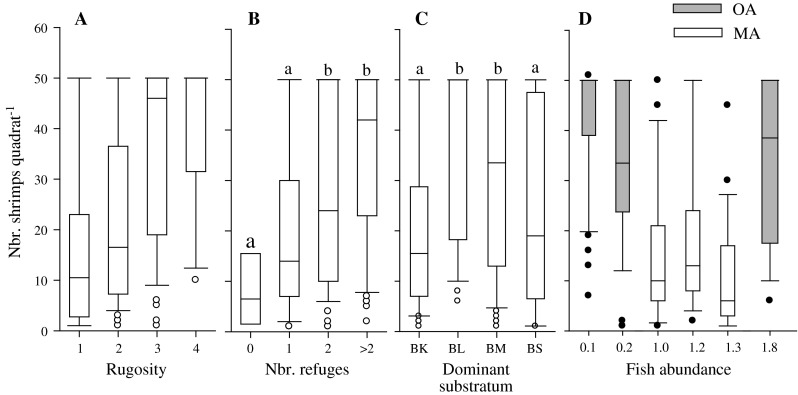
*R. typus* abundance in function of **a** rugosity, **b** number of refuges, **c** type of dominant substratum and **d** fish abundance and management status. The median is the *line crossing the* *box*; the boundaries of the box represent the 25 and 75 ‰; the whiskers are the minimum and maximum within 1.5 times the interquartile range; outliers are indicated by *open and filled circles*; *N* = 220. The *letters above the bars* indicate significant differences (*P* < 0.05) in shrimp abundance between categories

The best-fitting GEE model indicated that the abundance of *R. typus* was significantly influenced by rugosity, number of refuges, type of dominant substratum and the interaction between site management status and fish abundance (Table 2B). Neither the presence of urchins, the intensity of predation, nor the abundance of fish or its interaction with rugosity influenced shrimp abundance. Median shrimp abundances did not differ between quadrats with rugosity categories 1 and 2, nor between quadrats with rugosity 3 and 4 (Fig. 3a; Table S3). Shrimp abundance was three times higher in quadrats with rugosity categories 3 and 4 pooled together than in quadrats with rugosity 1 and 2 pooled together (*U* = 3,189.5, *N*_1–2_ = 113, *N*_3–4_ = 102, *P* < 0.001). Median shrimp abundance did not differ between quadrats with 0 and 1 refuge, but increased with higher number of refuges (Fig. 3b; Table S3) and, overall, was almost 4 times greater in quadrats containing >1 refuges. Shrimp abundance did not differ between quadrats dominated by bedrock, and those dominated by small boulders, but was approximately 2 times greater in quadrats dominated by large boulders and 3 times greater in those dominated by medium boulders (Fig. 3c; Table S3). Shrimp abundance was negatively related to fish abundance in managed areas (estimated regression coefficient of the full GEE model: b = −1.68 ± SE 0.13, $\chi_{1}^{2}$ = 166.87, *P* < 0.001; Fig. 3d), but not in open-access areas (b = 0.37 ± SE 0.03, $\chi_{1}^{2}$ = 1.26, *P* = 0.26).

**Fig. 4:**
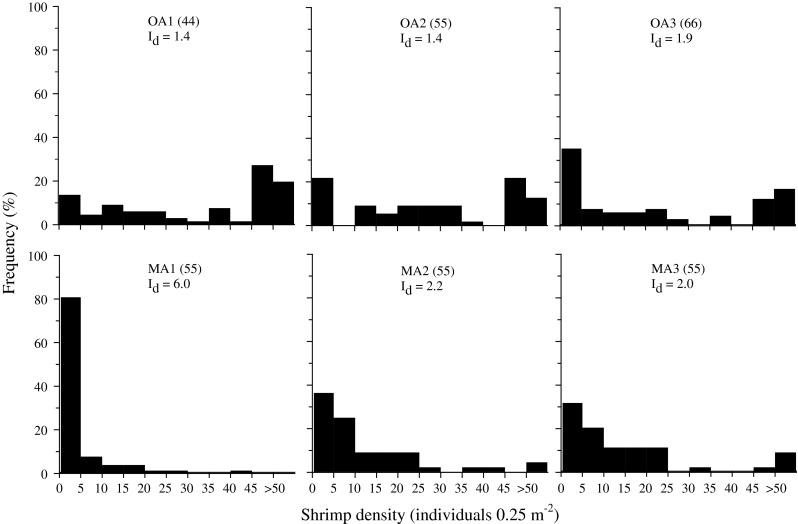
Frequency of occurrence of *R. typus* per quadrat at the six studied sites with the corresponding Morisita’s index of dispersion, *I*_*d*_. *I*_*d*_ > 1 indicates a pattern of aggregated dispersal

*R. typus* was aggregated at all sites (Morisita’s Index, *I*_*d*_ > 1; chi-square test, *P* < 0.001). Shrimps were patchiest at MA1 (*I*_*d*_ = 6.0), where >80 % of the quadrats were without shrimps and the least patchy at sites inside La Herradura bay (*I*_*d*_ = 1.4 at OA1 and OA2; Fig. 4), where 14 and 21 % of the quadrats lacked shrimps. Large aggregations of >45 individuals were more frequently observed at OA1, OA2 and OA3 (29.2–37.5 % of observations) than at MA2 or MA3 (<12 %). MA1 had only 3 aggregations with 20–45 individuals.

**Fig. 5:**
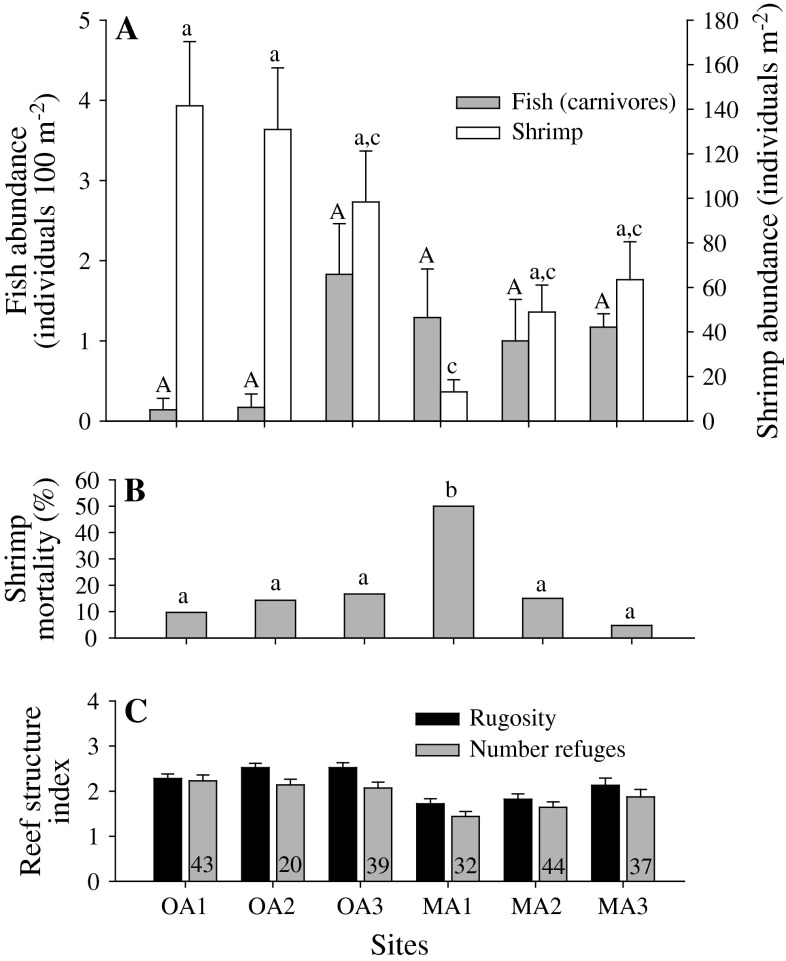
**a** Mean abundance of fish and *R. typus* (±95 % confidence intervals) at six different reef barrens in northern-central Chile. *Different letters* indicate significant differences (*P* < 0.01) among sites. Sample sizes: OA1 = 4, OA2 = 5, OA3 = 6, MA1 = 5, MA2 = 5 and MA3 = 5. **b** Mortality of tethered shrimps after 30 min in the field. *Different letters* indicate a significant difference between sites (*P* < 0.05). Sample sizes: OA1 = 31, OA2 = 21, OA3 = 18, MA1 = 20, MA2 = 20 and MA3 = 21. **c** Mean (±95 CI) reef structure index (mean of rugosity and number of refuge categories) for each barrens site. *Numbers* indicate the percentage of large refuges (>15 cm aperture) out of all refuges available per site

Among all sites, shrimps more often occupied large refuges than small ones ($\chi_{2}^{2}$ = 23.34, *N* = 267, *P* < 0.001), despite the fact that their availability ranged from 20 % (OA2) to 44 % (MA2) of all refuges available at a site. Only at MA1 shrimps used large and small refuges according to their availability ($\chi_{2}^{2}$ = 0.390, *N* = 48, *P* = 0.532). The availability of large refuges did not vary significantly (Kruskall–Wallis test: *H*_5_ = 4.20, *P* = 0.52; Fig. 5) among sites.

### Intersite differences between fish and shrimp abundances and predation rates

The abundance of *R. typus* per transect varied between sites, from ~10 individuals m^−2^ at MA1 to >100 individuals m^−2^ at OA1 and OA2 (*H*_5_ = 20.61, *P* = 0.001; Fig. 5). The abundance of *R. typus* varied also between sites when empty quadrats were removed from the analysis, from ~40 individuals m^−2^ at MA1 to >160 individuals m^−2^ at OA1 (*H*_5_ = 2.14, *P* < 0.001). Mean shrimp abundances per site were higher in OAs than in MAs (Mann–Whitney *U* test, *U* = 31.0, *N*_1_ = 3, *N*_2_ = 3, *P* < 0.001). Less than 2 % of the shrimps at any site were observed on open rock surfaces, with the rest in refuges under boulders or in rock crevices of variable sizes.

At the scale of the transect, the abundance of predatory fishes (five species; Table 3) varied from ~0.1 individuals 100 m^−2^ (OA1 and OA2) to almost two individuals 100 m^−2^ (OA3). Fish sizes did not vary among sites (*H*_5_ = 4.98, *P* = 0.42). Although no significant difference was found in fish abundances among sites (respectively, *H*_5_ = 9.83, *P* = 0.08 and), nor between open-access areas (0.7 individuals m^−2^ ± 0.6 SE) and managed areas (1.2 individuals m^−2^ ± 0.1 SE; *U* = 3.0, *N*_1_ = 3, *N*_2_ = 3, *P* = 0.71; Fig. 5), fish abundances were the lowest at OA1 and OA2 compared to the managed areas (Fig. 5). GEE models confirmed that the abundance of predatory fishes was not influenced by management status, neither by rugosity nor the interaction between these two factors (Table 2C). With the exception of MA1, where 50 % mortality was recorded after 30 min, mortality did not vary between the other five sites (Fig. 5), where it ranged from 5 to 17 % (mean, 12.1 % ± 2.8 SE). Among all sites, shrimp mortality was not correlated with fish abundance (*r*_*s*_ = 0.60, *N* = 6, *P* = 0.208), rugosity (*r*_*s*_ = −0,261, *N* = 6, *P* = 0.618) or refuge number (*r*_*s*_ = −0.493, *N* = 6, *P* = 0.321). Two video-recordings of the tethering experiments at MA1 and OA3 showed the shrimps being attacked by a group of *Scartichthys viridis* (Blenniidae). The abundance of this fish species (2.1 ± 0.50 SE individuals 100 m^−2^) did not differ among the six sites (Kruskal–Wallis test: *H*_5_ = 9.25, *P* = 0.10).

**Table 3:** Abundance (individuals 100 m^−2^ ± SE) and sites of observation of predatory fishes observed during the visual census

Fish species (sample size)	Visual census		Stomach contents				
	Abundance	Sites	TL	*N*	*R. typus*		
					% F	No	Size
*Cheilodactylus variegatus* (Cheilodactylidae) (5)	3.0 ± 1.5	OA1, OA3, MA1, MA2, MA3	41–55	10	10	1.0	17.2
*Pinguipes chilensis* (Pinguipedidae) (14)	1.5 ± 1.0	OA1, OA3, MA3	45–64	25	60	2.7 ± 0.5	13.4
*Paralabrax humeralis* (Serranidae)	0.8 ± 0.5	OA2, MA2, MA3	40–50	3	67	2.5 ± 0.5	13.7
*Seriola lalandi* (Carangidae)	0.3 ± 0.3	MA2		0	0		
*Hippoglossina macrops* (Paralichthyidae)	0.2 ± 0.2	MA3		0	0		
*Graus nigra* (Kyphosidae)	0		50–81	8	0	9.9 ± 0.3	10.0
*Semicossyphus darwini* (Labridae)	0		47–58	4	25	1.0	8.0
*Genypterus chilensis* (Ophidiidae)	0		58–96	4	100	1.8 ± 0.5	13.7

TL (minimum–maximum, in cm) and total number of non-empty stomachs (N) of fishes captured at OA3, MA1 and MA2. Frequency of occurrence (% F), mean number (±SE) of shrimps per stomach (individuals stomach^−1^ ± SE) and median size (mm) of *R. typus* found in stomachs

### Shrimp sizes in their natural habitat and in fish stomachs

**Fig. 6:**
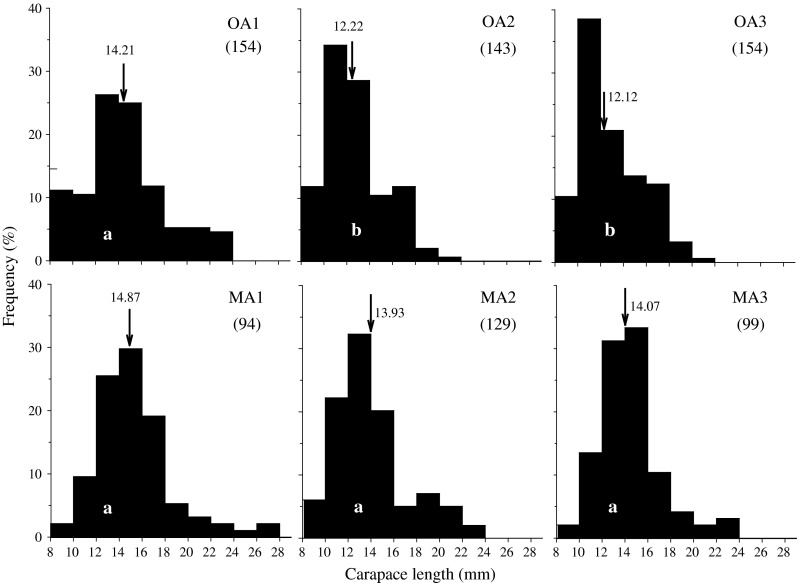
*R. typus* size distribution at the six reef barrens in Chile. Median shrimp size is indicated by an *arrow*. *Different letters* indicate significant differences between the medians (*P* < 0.001, sample size within brackets)

The median size of *R. typus* captured in the natural habitat was similar at OA2 and OA3, but differed from all the other sites (Fig. 6). The largest individuals at OA2 (21.6 mm) and at OA3 (20.1 mm) were smaller than at the other sites. The largest shrimp (27.2 mm) was found at MA1.

A total of 55 individuals of seven predatory fish species were captured at OA3, MA1 and MA2 (Table 3); 23 (42 %) of stomachs had at least one *R. typus*, with 2.4 ± 0.3 SE individuals stomach^−1^. All *Genypterus chilensis* (*N* = 4) and 2 out of 3 *Paralabrax humeralis* had consumed at least one shrimp. Of those species that were among the most abundant predators observed in the field, 15 out of the 25 (60 %) *Pinguipes chilensis* and 1 out of 10 *Cheilodactylus variegatus* had at least one shrimp in their stomach (Table 3). There was no correlation between the size of the predators and the number (*r*_*s*_ = 0.13, *N* = 23, *P* > 0.10) nor the size (*r*_*s*_ = −0.58, *N* = 23, *P* = 0.79) of shrimps eaten.

**Fig. 7:**
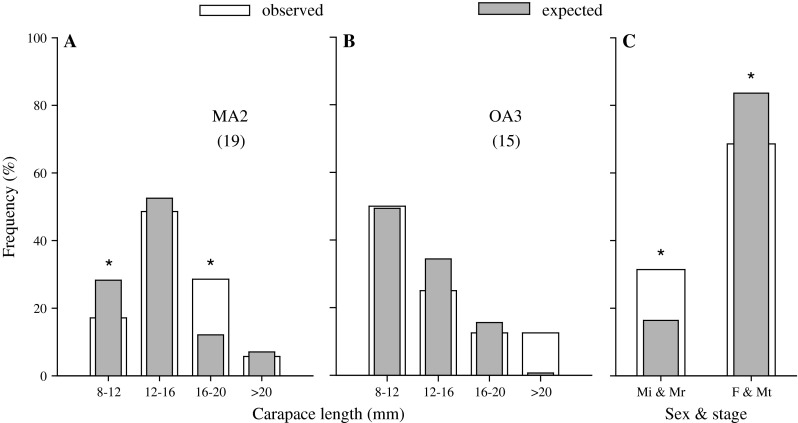
**a** Size distribution of *R. typus* in fish stomachs and in the field at **a** MA2 and **b** OA3 (sample size within brackets), and frequency **c** sex and ontogenetic stage (Mi = intermedius male, Mr = robustus male, F = females and Mt = typus males) at the two sites pooled together. An *asterisk* indicates a significant difference between the frequencies (*P* < 0.05)

Mean size of *R. typus* from all fish stomachs was 13.9 ± 0.6 mm. The size distribution of these shrimps was different from that observed in the field at MA2 ($\chi_{3}^{2}$ = 9.75, *N* = 15, *P* = 0.02; Fig. 7a), and large shrimps (16–20 mm) were more frequently eaten than smaller ones ($\chi_{1}^{2}$ = 6.01, *P* = 0.01; Fig. 7a). However, no such difference was found at OA3 ($\chi_{3}^{2}$ = 0.78, *N* = 19, *P* = 0.94; Fig. 7b) although there was a tendency for fishes to eat greater proportions of shrimps >20 mm than were observed in the field. Nonetheless, pooled together across MA2 and OA3 and shrimp categories, robustus and intermedius males were consumed by fishes more often than typus males or females relative to their frequency in the field ($\chi_{1}^{2}$ = 5.77, *N* = 35, *P* = 0.02; Fig. 7c).

## Discussion

As expected, at the microhabitat scale within the reef, the complexity of the habitat (i.e. substratum structure and number of refuges) influenced both the presence and abundance of *R. typus*. However, in contrast to our predictions, shrimps tended to aggregate in large crevices providing less protection against predators rather than occupy small refuges. Predation tended to affect the spatial distribution of the shrimps within the reef but not overall shrimp abundances. Shrimp abundance was negatively related to fish abundance in managed areas, but not in open-access areas, which we attribute to higher levels of predation upon shrimps at these sites. Shrimp body-size distribution in the field was unaffected by the apparent preference of fish for larger prey individuals. We did not find any significant effect of site management status on fish abundance, nor any effect of reef structure. However, predatory fish were relatively scarce in two of the three open-access areas where shrimp abundances were highest. The lack of apparent top-down effects of fish on shrimps could be due to overall low fish abundances as discussed below.

### Relationship between shrimps and fishes

We recorded shrimp densities that were substantially higher (up to ten times greater) than a 1994 study by Caillaux and Stotz (2003) at the open-access areas OA2 and OA3 (L Caillaux, pers comm), which may be a consequence of reduced direct predation on shrimps. Indeed, the abundance and the size of the most important fish predators (e.g. *Semicossyphus darwini*, *Pinguipes chilensis*, *Hemilutjanus macrophthalmos*, *Cheilodactylus variegatus*) that feed on *R. typus* (Table 3) have been dramatically reduced by overfishing during the last two decades (SERNAPESCA 1979–2008; Godoy et al. 2010). We could not detect any significant difference in fish abundance between managed areas and open-access sites, probably because of the high fish abundances recorded at OA3. This site is relatively close to management areas, and it is possible that highly mobile predatory fishes roam into areas with high shrimp abundances. It is nonetheless notable that, in our study, shrimp abundances within the managed areas were lower than in the open-access areas, and this is consistent with higher predation risk and more fishes within these managed areas. There is also direct and indirect evidence for the importance of fish predation in some of the MAs. For example, at MA1 shrimp, abundances were similar in 1994 (L Caillaux pers comm) and in our study (~10 individuals m^−2^ in 1994 and ~18 individuals m^−2^ in 2010), and these were the lowest recorded at any of our six study sites.

The intensity of predation was surprisingly higher at MA1 compared to all other sites. The actual intensity of predation may have been overestimated by opportunistic attacks of the groups of *S. viridis* (Blenniidae), a fish species commonly considered to be an herbivore (Muñoz and Ojeda 2000). However, densities of *S. viridis* were similar among all six sites, suggesting that the much higher predation at MA1 cannot solely be attributable to the presence of this blenny.

Tethered shrimps could not hide on the PVC plates, which were placed on the reef surface in places where they were fully exposed to visual predators. We are therefore confident that all tethered shrimps were visible to predatory fishes and that differences in reef topography among sites did not affect shrimp detectability. This assertion is supported by the fact that we detected no relationship between habitat structure (rugosity and number of refuges), fish abundance and shrimp mortality.

### Influence of habitat structure on shrimp abundance and distribution

The positive influence of habitat complexity on shrimp densities recorded in our study is consistent with results of other studies that described the highest diversity and abundance of subtidal communities associated to the most complex reefs (review by Knudby et al. 2007). *R. typus* was frequently absent on substrata of limited complexity, probably because of the prohibitive risks of predation associated with these habitats (Herrnkind and Butler 1986; Wahle and Steneck 1992). On the other hand, shrimp presence did not differ between the highest categories of reef rugosity and refuge availability, which may indicate that predation risk for shrimps on shallow barrens is not sufficiently intense to create a difference in probability of survival beyond a critical threshold of refuge availability. Similarly, Primavera (1997) showed that fish predation on the mangrove-associated penaeid shrimps was higher over bare sand than among pneumatophores, but did not vary with an increasing density of pneumatophores (see also Canion and Heck 2009).

*R. typus* occurred more often and in higher abundance on substrata (large and medium boulders), providing crevices or holes with large apertures that fishes can access, thus offering limited protection. They did so even when small refuges were available. This is surprising at first, because vulnerable prey should hide in refuges that match their body-size, as shown for small juvenile spiny lobsters (e.g. Eggleston et al. 1990, 1997). This result, however, is in agreement with the interpretation that overall predation risk for *R. typus* in our study area has been low (see above), allowing shrimps to occupy large shelters that offer limited protection. On the other hand, shrimps did not prefer large over small refuges and used all the refuges available at MA1, where predation risk was the highest. Social interactions or predation risk may further modify choice of refuge size. For example, spiny lobsters aggregate in large refuges in preference to smaller ones when predation risk is low and conspecific density high (Eggleston and Lipcius 1992; Childress and Herrnkind 1997). The low level of predation and the high density of *R. typus* on shallow barrens may also have favoured large group of shrimps to aggregate in shelters widely open. Indeed, large aggregations were more frequent at sites where fish abundance and, presumably, predation rates were lowest, and shrimp abundance was highest (OA1 and OA2); conversely, shrimps were least numerous at MA1. Although aggregated at all sites, *R. typus* distribution was the patchiest at MA1 and less patchy (i.e. tending to random) at OA1 and OA2. Similarly, Spieler (2003) demonstrated that frog tadpoles aggregate in shallow water of savannah ponds during the day, when predation risk is high, and swim randomly at night, when predation risk decreased. This, in combination with our findings, suggests that the intensity of predation can induce changes in prey behaviour, leading to adjustments in their refuge use and spatial distribution.

However, it must be stressed that our study was not designed to test the factors influencing the gregarious distribution of the shrimps. Nonetheless, *R. typus* may be a good model to investigate the factors that favour the evolution of gregarious behaviours of small vulnerable prey.

### Direct effects of predation on body-size structure of shrimps

Our results indicate that the size of *R. typus* found in fish stomachs was independent of the size of the predator, with a dominance of large *R. typus* (Fig. 7). This contrasts with previous studies that demonstrated a relationship between fish size and invertebrate prey size (e.g. Edgar et al. 1994; Edgar and Shaw 1995b). In our study, the fish predators, irrespective of their size, tended to consume more large shrimps *R. typus*, and more robustus and intermedius males than should be expected from their distribution in the field. Large males may be more exposed to predation because they take more risks during mating interactions (Correa and Thiel 2003; Van Son and Thiel 2006) or to access food (Arana and Henríquez 1983). Although robustus males competing for access to females show similar searching activity as typus males (Dennenmoser and Thiel 2006), the time robustus males spend guarding mated females is not reduced in the presence of a predator (Van Son and Thiel 2006). Robustus males may consequently suffer more from predation than small—less preferred—individuals. As a result, the largest shrimps do not reach sizes at which fish predation is significantly reduced, as observed in lobsters (Wahle and Steneck 1992). Future studies are needed to test whether predation risk increases with shrimp body size; fishes may preferably consume large males, or behavioural differences between large and small males may expose the former to higher predation risks. These two explanations may not be mutually exclusive.

Strong direct effects of predators on large prey individuals should skew body-size distributions towards smaller individuals (Edgar and Shaw 1995b), but we did not observe this for *R. typus* population structures, perhaps because the overall predation rates at most of our study sites was low (see above). Body-size distributions of *R. typus* collected in Valparaiso (Arana and Henríquez 1983), and with an air-lift sampler at OA1 in October 1999 by Correa and Thiel (2003), were similar to those reported in the present study. Thus, while body-size distribution of *R. typus* varies seasonally (Correa and Thiel 2003), population structure appears to be relatively stable among years and at different locations.

## Concluding remarks

Although we found no evidence for direct effects of predation on shrimp population structure nor on shrimp abundance inside open-access areas, fish abundance was inversely related to reduced shrimp abundance in managed areas. In addition, the lowest abundances of predatory fish and the highest shrimp abundances were recorded in two of the three open-access areas, and the highest shrimp mortality was in one of the managed areas where fishing was restricted. Further investigation of potential top-down effects of predators on mesoconsumers such as *R. typus* in managed versus open-access areas would be of value to predict changes caused by population declines of top predators (Heithaus et al. 2008; Eriksson et al. 2011), especially given the important role of this shrimp in structuring benthic communities in Chilean waters (Dumont et al. 2009, 2011a, b).

## Electronic supplementary material

Below is the link to the electronic supplementary material.

Supplementary material 1 (DOCX 23 kb)
