# Supplementary material for: Effects of predation and habitat structure on the abundance and population structure of the rock shrimp Rhynchocinetes typus (Caridea) on temperate rocky reefs
Source: Mar Biol. 2012 Jul 13;159(9):2075–89. doi: 10.1007/s00227-012-1994-6 (PMC3873050; doi:10.1007/s00227-012-1994-6)
Supplement: Supplementary file 1 — Supplementary material 1 (DOCX 23 kb) [file 227_2012_1994_MOESM1_ESM.docx]

# ELECTRONIC SUPPLEMENTS

Table S1. Correlations between *R. typus* size (expressed below as carapace length, CL) and telson length (TeL), the first segment of the 3^rd^ maxilliped (ML) of females and typus males and the first appendage of the 1^st^ pereopod, i.e. chelae (PL) of shrimp found in fishes stomachs.

| **Appendages compared** | **Ratio**  **(mean ± SE)** | **Spearman correlation (P < 0.001)** | **Sex and ontogenetic stage of shrimp measured (N)** |
| --- | --- | --- | --- |
| RL vs. CL | 0.76 ± 0.011 | r _=_ 0.89 | All individuals (57) |
| TeL vs. CL | 0.52 ± 0.004 | r _=_ 0.97 | All individuals (47) |
| ML vs. CL | 0.70 ± 0.004 | r = 0.90 | Females, typus males (515) |
| PL vs. CL | 0.4 ± 0.03 | r = 0.75 | Females, typus males (515) |
| PL vs. CL | 0.83 ± 0.13* | r _=_ 0.85 | robustus and intermedius males (90) |

* robustus and intermedius males can only be differentiated by comparing the PL/CL ratio ([Correa et al. 2000](#_ENREF_17)). For shrimp found in fish stomachs, these two ontogenetic stages were defined from the presence of hair on chelae, but could not be differentiated. We thus calculated CL using the average of the ratios PL/CL _intermedius males =_ 0.64, SE = 0.08, N = 53 and PL/CL_robustus males =_ 1.02, SE = 0.18, N = 47.

Table S2. Statistical values of the chi-square tests comparing the frequency of shrimp being present in the quadrats (observed) with the frequency expected by chance (p = 0.5; see text and Fig. 2).

| **Responses variables** | **N** | **df** | **Chi-square** | **P** |
| --- | --- | --- | --- | --- |
| Rugosity 1 | 22 | 1 | 6.31 | 0.012 |
| Rugosity 2 | 96 | 1 | 38.76 | < 0.001 |
| Rugosity 3 | 85 | 1 | 81.00 | < 0.001 |
| Rugosity 4 | 17 | 1 | 11.44 | < 0.001 |
| 0 refuge | 7 | 1 | 43.84 | < 0.001 |
| 1 refuge | 74 | 1 | 80.00 | < 0.001 |
| 2 refuges | 70 | 1 | 64.22 | < 0.001 |
| > 2 refuges | 69 | 1 | 53.12 | < 0.001 |
| Bedrock | 64 | 1 | 1.67 | > 0.10 |
| Large boulders | 55 | 1 | 31.34 | < 0.001 |
| Medium boulders | 76 | 1 | 73.05 | < 0.001 |
| Small boulders | 20 | 1 | 12.57 | < 0.001 |
| Fish abundance (< 1 individual 100m^-2^) | 89 | 1 | 55.68 | < 0.001 |
| Fish abundance (1 individual 100m^-2^) | 164 | 1 | 11.14 | < 0.001 |
| Fish abundance (2 individual 100m^-2^) | 65 | 1 | 5.48 | 0.019 |
| Shrimp morality < 10 % | 89 | 1 | 56.02 | < 0.001 |
| Shrimp morality 11-15 % | 164 | 1 | 39.02 | < 0.001 |
| Shrimp morality > 15 % | 65 | 1 | 5.48 | 0.019 |

Table S3. Statistical values of the Dunn’s tests comparing shrimp abundances in nonempty quadrats between each category of rugosity, number of refuges and type of dominant substratum. Number within brackets is the sample size of the corresponding category.

| **Response variables** | **Comparisons** | **df** | **Dunn’s test** | **P** |
| --- | --- | --- | --- | --- |
| Rugosity | 1(22) - 2(96) | 1 | 1.60 | 0.66 |
|  | 1 - 3(85) | 1 | 4.54 | < 0.001 |
|  | 1 - 4(17) | 1 | 4.04 | < 0.001 |
|  | 2 - 3 | 1 | 4.76 | < 0.001 |
|  | 2 - 4 | 1 | 3.53 | 0.003 |
|  | 3 - 4 | 1 | 0.85 | > 0.5 |
| Number of refuges | 0(7) - 1(75) | 1 | 1.97 | 0.29 |
|  | 0 - 2(70) | 1 | 2.89 | 0.02 |
|  | 0 - > 2(69) | 1 | 4.16 | < 0.001 |
|  | 1 - 2 | 1 | 2.49 | 0.04 |
|  | 1 - 3 | 1 | 5.28 | < 0.001 |
|  | 2 - > 2 | 1 | 2.87 | 0.02 |
| Dominant substratum | Bedrock(64) - large boulders(55) | 1 | 4.52 | < 0.001 |
|  | Bedrock - medium boulders(76) | 1 | 3.48 | 0.003 |
|  | Bedrock - small boulders(20) | 1 | 0.65 | > 0.5 |
|  | Large boulders - medium boulders | 1 | 1.43 | > 0.5 |
|  | Large boulders - small boulders | 1 | 3.38 | 0.002 |
|  | Medium boulders - small Boulders | 1 | 2.51 | 0.04 |
